# Low-dose chloroquine treatment extends the lifespan of aged rats

**DOI:** 10.1007/s13238-021-00903-1

**Published:** 2022-01-12

**Authors:** Wei Li, Zhiran Zou, Yusheng Cai, Kuan Yang, Si Wang, Zunpeng Liu, Lingling Geng, Qun Chu, Zhejun Ji, Piu Chan, Guang-Hui Liu, Moshi Song, Jing Qu, Weiqi Zhang

**Affiliations:** 1 Advanced Innovation Center for Human Brain Protection, National Clinical Research Center for Geriatric Disorders, Xuanwu Hospital Capital Medical University, Beijing, 100053 China; 2 State Key Laboratory of Membrane Biology, Institute of Zoology, Chinese Academy of Sciences, Beijing, 100101 China; 3 State Key Laboratory of Stem Cell and Reproductive Biology, Institute of Zoology, Chinese Academy of Sciences, Beijing, 100101 China; 4 CAS Key Laboratory of Genomic and Precision Medicine, Beijing Institute of Genomics, Chinese Academy of Sciences, Beijing, 100101 China; 5 China National Center for Bioinformation, Beijing, 100101 China; 6 Institute for Stem Cell and Regeneration, Chinese Academy of Sciences, Beijing, 100101 China; 7 University of Chinese Academy of Sciences, Beijing, 100049 China; 8 Aging Translational Medicine Center, Xuanwu Hospital, Capital Medical University, Beijing, 100053 China; 9 Beijing Institute for Stem Cell and Regenerative Medicine, Beijing, 100101 China; 10 Sino-Danish College, University of Chinese Academy of Sciences, Beijing, 101408 China; 11 Sino-Danish Center for Education and Research, Beijing, 101408 China; 12 Chongqing Renji Hospital, University of Chinese Academy of Sciences, Chongqing, 400062 China

## Dear Editor,

Chloroquine (CQ) has long been used as an anti-malarial agent (Wellems and Plowe, 2001). Recently, CQ has also been applied to treat viral infection and related diseases (Wellems and Plowe, 2001; Huang et al., 2020). However, the safety and efficacy of its applications are still under extensive debate (Solomon and Lee, 2009). Here, we discovered that low-dose CQ has a geroprotective effect on physiologically aged rats. Low-dose CQ prolonged lifespan, repressed systemic inflammation, and inhibited fibrosis across multiple tissue types in aged rats. Furthermore, we constructed transcriptomic maps for 6 tissues (kidney, small intestine, liver, heart, lung, and aorta) upon CQ treatment, thus revealing the effects of CQ at a systemic level. CQ treatment mitigated age-related molecular changes and repressed genes linked to fibrosis and the inflammatory response. Altogether, our data provide a valuable resource for investigating the impact of CQ on multiple aged tissues, which may facilitate the development of clinical applications that mitigate age-related changes in the elderly.

CQ has been used to combat malaria for decades (Wellems and Plowe, 2001). As one kind of aminoquinoline drugs, CQ displays several advantages: easily absorbable, simple to synthesize, high efficacy, straightforward to use, and generally affordable (Solomon and Lee, 2009; Huang et al., 2020). CQ is used to treat a range of diseases, including inflammatory diseases, infectious diseases, and even multiple types of cancer (Solomon and Lee, 2009). CQ also alleviates the symptoms of rheumatoid arthritis, indicating its anti-inflammation property (Sargiacomo et al., 2020; Schrezenmeier and Dorner, 2020). In addition, studies increasingly indicate that CQ treatment is involved in the regulation of cellular senescence (Sargiacomo et al., 2020). CQ treatment results in a decreased ratio of cells that are positive for senescence-associated beta-galactosidase (SA-β-gal) (Sargiacomo et al., 2020), a well-known senescent marker (Cai et al., 2020). However, whether CQ treatment attenuates systemic aging and extends the lifespan of naturally aged individuals is unclear.

Despite the beneficial effects of CQ listed above, side effects have also been reported, eliciting widespread concerns. For example, high-dose CQ increases the risk of cardiac arrhythmia with cardiotoxicity (Wozniacka et al., 2006). Side effects have also been observed in other tissues, such as gastrointestinal tissues and the liver (Solomon and Lee, 2009). Notably, these side effects depend on the dose, administration method, and the duration of treatment (Solomon and Lee, 2009). Therefore, it is essential to carefully evaluate the side effects of CQ treatment to ensure safety at both tissue and organismal levels.

**Figure 1:**
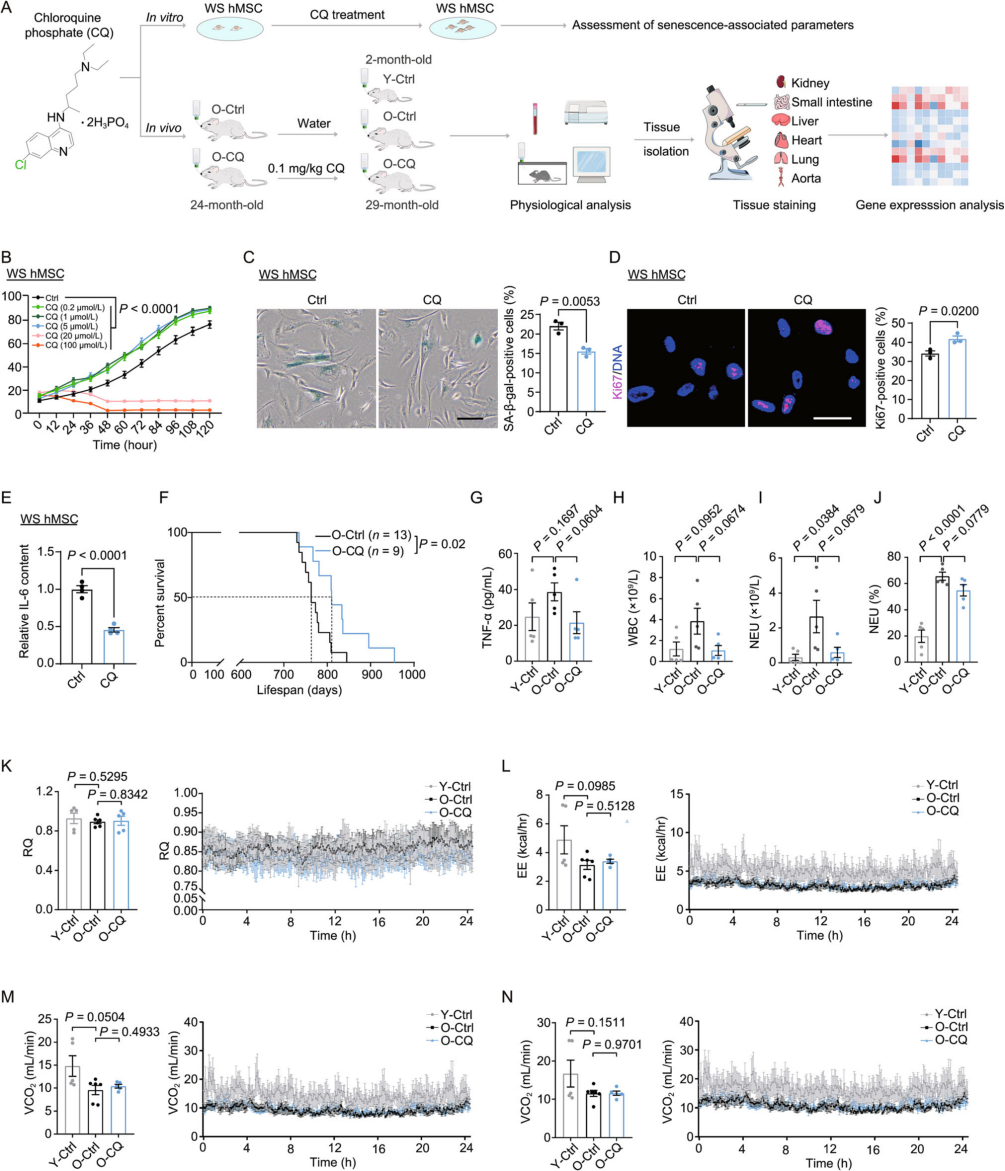
The geroprotective effect of CQ on cellular senescence and tissue aging. (A) Schematic diagram showing analysis of the CQ effectiveness *in vitro* and *in vivo*. Upper, the determination of an optimal concentration of CQ for a geroprotective role in WS hMSCs. Lower, the treatment of 24-month-old rats with drinking water (O-Ctrl) or CQ (O-CQ) for five months. Y-Ctrl, 2-month-old rats with drinking water. (B) Relative cell proliferation analysis upon CQ treatment at given concentrations in WS hMSCs (passage 7) by using the IncuCyte S3 live cell imaging system. The data are shown as means ± SD. *n* = 6 biological replicates per group. (C) SA-β-gal staining of CQ-treated and control WS hMSCs (passage 7). Scale bar, 100 μm. The data are shown as means ± SEM. *n* = 3 biological replicates per group (unpaired Student’s *t*-test). (D) Immunostaining of Ki67 in CQ-treated and control WS hMSCs (passage 7). Scale bar, 25 μm. Data are shown as means ± SEM. *n* = 3 biological replicates per group (unpaired Student’s *t*-test). (E) ELISA of IL-6 in CQ-treated and control WS hMSCs (passage 7). The data are shown as means ± SEM. *n* = 4 per group (unpaired Student’s *t*-test). (F) Survival curves for O-Ctrl (*n* = 13 rats) and O-CQ (*n* = 9 rats) rats (Log-rank test). (G) ELISA of TNF-α in the plasma of Y-Ctrl, O-Ctrl and O-CQ rats. The data are shown as means ± SEM. *n* = 5 rats per group (unpaired Student’s *t*-test). (H) The number of white blood cells (WBC) in the blood. The data are shown as means ± SEM. *n* = 5 rats per group (unpaired Student’s *t*-test). (I) The number of neutrophils (NEU) in the blood. The data are shown as means ± SEM. *n* = 5 rats per group (unpaired Student’s *t*-test). (J) The proportion of neutrophils (NEU) in the blood. The data are shown as means ± SEM. *n* = 5 rats per group (unpaired Student’s *t*-test). (K) Real-time measurement of respiratory quotient (RQ) of Y-Ctrl, O-Ctrl and O-CQ rats by metabolic cage detection. Right, representative curves of RQ measured within 24 h. The data are shown as means ± SEM. *n* = 5–6 rats per group (unpaired Student’s *t*-test). (L) Real-time measurement of energy expenditure (EE, kcal/h) of Y-Ctrl, O-Ctrl and O-CQ rats by metabolic cage detection. Right, representative curves of EE measured within 24 h. The data are shown as means ± SEM. *n* = 5–6 rats per group (unpaired Student’s *t*-test). (M) Real-time measurement of carbon dioxide emission rate (VCO_2_, mL/min) in Y-Ctrl, O-Ctrl and O-CQ rats by metabolic cage detection. Right, representative curves of carbon dioxide emission measured within 24 h. The data are shown as means ± SEM. *n* = 5–6 rats per group (unpaired Student’s *t*-test). (N) Real-time measurement of oxygen consumption rate (VO_2_, mL/min) of Y-Ctrl, O-Ctrl and O-CQ rats by metabolic cage detection. Right, representative curves of oxygen consumption measured within 24 h. The data are shown as means ± SEM. *n* = 5–6 rats per group (unpaired Student’s *t*-test)

To evaluate the impact of CQ on senescence, we treated human mesenchymal stem cells (hMSCs), multipotent stem cells present in most tissues, with the drug. We first tested a wide range of concentrations from 0.2 μmol/L to 100 μmol/L of CQ in Werner syndrome-specific mesenchymal stem cells (WS hMSCs) (Fig. 1A), a stem cell model for premature aging (Wu et al., 2018). We found that CQ promoted cell self-renewal at lower concentrations (0.2–5 μmol/L), but inhibited cell proliferation at higher concentrations (20 μmol/L or above) (Fig. 1B). Specifically, treatment with 1 μmol/L CQ decreased the percentage of SA-β-gal-positive cells and increased the number of Ki67-positive cells in WS hMSCs (Fig. 1C and 1D). Additionally, low-dose CQ treatment decreased IL-6 secretion of WS hMSCs (Fig. 1E). Our previous work indicated that the loss of heterochromatin was associated with the senescence of WS hMSCs (Wu et al., 2018; Bi et al., 2020), and we found low-dose CQ treatment upregulated the levels of heterochromatin-associated marks including H3K9me3, HP1γ, and LAP2, indicating that CQ treatment restored the heterochromatin landscape in WS hMSCs (Fig. S1A). Collectively, our results suggest that low-dose CQ alleviates senescence in prematurely aged stem cells.

To further examine the systemic effects of CQ *in vivo*, we treated 24-month-old Sprague Dawley (SD) male rats with CQ twice a week for 5 months at a low dose of 0.1 mg/kg orally by water to avoid potential side effects (Fig. 1A). Low-dose CQ administration extended the lifespan of rats by approximately 6% in terms of median longevity and by about 13% in terms of maximum longevity (Fig. 1F). CQ-treated rats also tended to have decreased serum TNF-α levels and reduced the numbers of circulating white blood cells (WBC) and neutrophils (NEU) in old rats (Figs. 1G–J and S1B), suggestive of attenuated chronic inflammation. In addition, CQ-treated rats maintained comparable body weights and blood glucose levels to age-matched control rats (Fig. S1C). Metabolic cage analysis was used to track the metabolic activity of CQ-treated rats. Respiratory quotient, energy expenditure, carbon dioxide emission rate and oxygen consumption rate were largely unaffected by CQ treatment (Figs. 1K–N). Altogether, our results demonstrate that low-dose CQ extends rat lifespan without affecting standard physiological metrics reflecting metabolic health.

**Figure 2:**
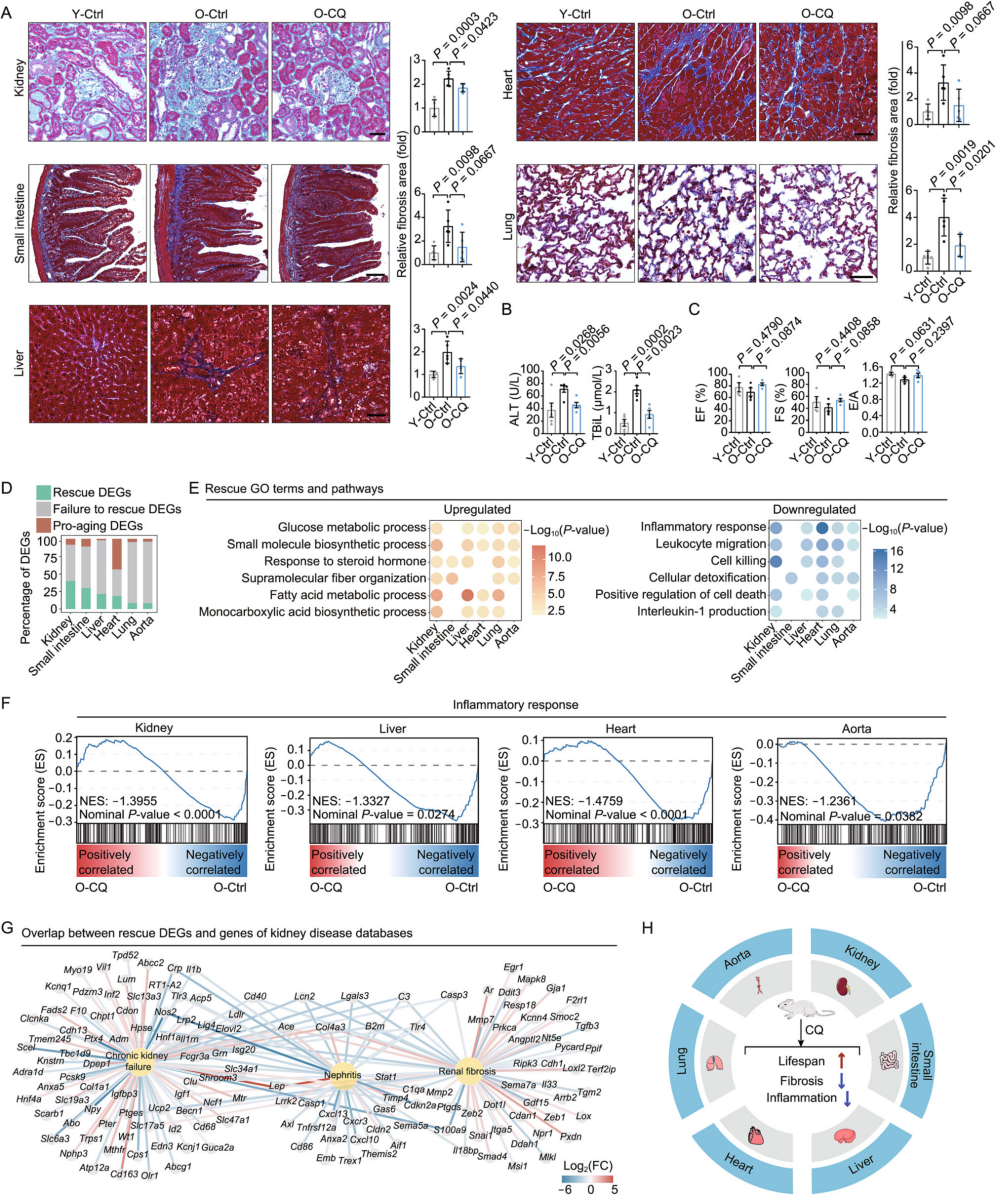
Changes in the transcriptional profiles of different tissues during aging and CQ treatment. (A) Masson staining of kidney, small intestine, liver, heart and lung, tissues from Y-Ctrl, O-Ctrl, O-CQ-treated rats. Scale bars, 60 μm for lung, 50 μm for liver and heart and 100 μm for kidney and small intestine. The data are shown as means ± SEM. *n* = 5 rats per group (unpaired Student’s *t*-test). (B) Alanine aminotransferase (ALT) and total bilirubin (TBiL) detection in the plasma. The data are shown as means ± SEM. *n* = 5 rats per group (unpaired Student’s *t*-test). (C) Left ventricular ejection fraction (EF), left ventricular fractional shortening (FS), and the ratio of peak velocity of early to late filling of mitral inflow (E/A) of Y-Ctrl, O-Ctrl and O-CQ rats. The data are shown as means ± SEM. *n* = 3–5 rats per group (unpaired Student’s *t*-test). (D) Bar plot showing the percentages of rescue DEGs, failure to rescue DEGs and pro-aging DEGs in the six tissues. Green indicates rescue DEGs, gray indicates a failure to rescue DEGs, and brown indicates pro-aging DEGs. (E) Representative GO terms and pathways enriched in rescue DEGs shared by at least three tissues. (F) Gene set enrichment analysis (GSEA) plots showing decreased inflammatory response in kidney, liver, heart and aorta upon CQ treatment. (G) Network plot showing rescue DEGs associated with renal diseases in kidney. (H) Schematic diagram illustrating the geroprotective effects of CQ in different tissues

To evaluate the effects of CQ on different tissues, we examined the effect of CQ in the kidney, liver, heart, small intestine, and lung, all of which are key organs that contribute to systemic aging. Generally, the tissue index (tissue weight divided by body weight) and morphological integrity were unaffected by CQ treatment despite the fact that the absolute weight of liver was decreased by CQ treatment (Figs. S1D, S1E and S2A). We inspected aging-related parameters in tissues using histological examination, fibrosis evaluation, aggresome staining and SA-β-gal staining (Geng et al., 2019). CQ treatment alleviated fibrosis in multiple aged tissues, such as kidney, liver, and lung (Fig. 2A). In addition, the number of CD68-positive macrophages and aggresome-positive cells in the liver (Figs. S2B and S2C), as well as levels of alanine aminotransferase (ALT) and total bilirubin (TBiL) in the blood were reduced after CQ treatment (Fig. 2B), suggesting that CQ has a liver-protective effect. However, CQ failed to reduce the levels of SA-β-gal positivity in most tissues, albeit with a decreasing trend in lung (Fig. S2D). Notably, the cardiac function seems not adversely affected by low dose of CQ treatment in aged rats (Fig. 2C). Collectively, these data suggest that the low-dose CQ treatment alleviates aging phenotypes in a tissue-specific manner without detectable cardiac side effects.

To uncover the effects of CQ on tissue-specific gene expression, kidney, small intestine, liver, heart, lung, and aorta tissues were collected from CQ-treated and control groups and subjected to high-throughput RNA sequencing along with 2-month-old rats as young controls (Fig. 2D). By evaluating the transcriptomes from young and old control rats, we identified aging-related differentially expressed genes “Aging DEGs” across multiple tissues (Figs. S3A and S3B). By comparing transcriptomes from CQ-treated and control old rats, we defined CQ-induced differentially expressed genes “CQ DEGs” across multiple tissues (Figs. S3A and S3B). Further exploration of the potential effects of CQ on aging-associated gene sets identified four groups of DEGs with distinct biological implications, i) aging DEGs that were reversed by CQ treatment, labeled “Rescue DEGs”, ii) aging DEGs that were unchanged upon CQ treatment, called “Failure to rescue DEGs”, iii) CQ-DEGs that were unchanged during aging, namely “CQ specific DEGs”, and iv) aging DEGs that were exacerbated after CQ treatment, namely “Pro-aging DEGs” (Fig. S3A). Notably, tissue specificity was observed in the number and distribution of these four types of DEGs, suggesting that CQ exhibits a tissue-dependent effect (Fig. S3B). Taken together, we established the transcriptomic landscape of multiple tissues in response to aging and CQ treatment in rats, which enables us to delineate the potential effects of CQ on aging in a tissue-specific manner.

Consistent with the observation that CQ treatment alleviated the signs of tissue aging and extended the lifespan of aged rats, CQ treatment rescued the expression of aging DEGs in tissues to varying extents (Fig. 2D). For instance, about 40% of genes in aged kidneys, 30% of genes in aged small intestines, and 20% of genes in aged livers were rescued by CQ treatment (Figs. 2D and S3A). These results suggest that CQ treatment mitigates aging-related molecular changes in a tissue-specific manner. The most significant dynamic changes in gene expression were observed in kidney, consistent with the phenotypic improvement described above (Fig. 2A). We systematically analyzed the “Rescue DEGs” using Gene Ontology (GO) terms across six tissues to uncover any common effects of CQ across multiple tissues. We found that the genes in the categories of glucose metabolic process, small molecule biosynthetic process, fatty acid metabolic process, and monocarboxylic acid biosynthetic process were upregulated in at least four tissues upon CQ treatment, and were reduced during aging (Fig. 2E). By contrast, genes associated with the inflammatory response, leukocyte migration, cell killing, and positive regulation of cell death, were increased during aging and were downregulated in at least four tissues upon CQ administration (Fig. 2E). Additionally, gene set enrichment analysis (GSEA) confirmed that CQ treatment attenuated the gene expression involved in the inflammatory response, IFN response, and senescence-associated secretory phenotype (SASP) in multiple aged tissues (Figs. 2F, S3C and S3D). IL-6-JAK-STAT3 and pro-fibrotic TGF-β signaling pathways were also downregulated by CQ treatment in aged livers (Figs. S3E and S3F).

We also performed an integrative comparative analysis of our transcription profiles with aging/longevity-associated genes from the Aging Atlas database (Aging Atlas, 2021). We found that the expression of aging-associated genes, such as *Cdkn2b*, *Cdkn2a*, and *Cdkn1a* were downregulated by CQ treatment (Fig. S3G). To unravel the systemic effects of CQ treatment, we further focused on those 76 “Rescue DEGs” that were shared by at least three tissues (Fig. S4A). Among these genes, the pro-inflammatory factors *S100a9*, *S100a8*, *Cxcl1*, and *Il1b* were decreased, and *Hsp90aa1*, a major molecular chaperon that maintains protein homeostasis and thereby plays a cytoprotective role, was increased in multiple tissues after CQ treatment (Fig. S4A). These results suggest that low-dose CQ treatment stimulates the upregulation of a systemic geroprotective transcriptional network across different tissues.

Our analysis revealed that the kidney showed the upregulation of the most rescue genes upon CQ treatment (Fig. 2D). This explains the prominent geroprotective effects of CQ treatment on kidney (Figs. 2D and S3B), indicating that low-dose CQ improves nephron functions, including metabolic processing of nutrients and removal of waste materials (Fig. S4B). In addition, GO terms such as response to virus, leukocyte mediated cytotoxicity, and immune effector process were repressed by CQ in aged kidneys (Fig. S4B). Consistently, rescue DEGs upregulated by CQ contained a series of kidney-specific organic anion transporters, such as *Slc17a2*, *Slc24a3*, *Slc31a1* and *Slc36a4* (Fig. S4C), which play vital roles in regulating anion balance in the body. Furthermore, CQ treatment attenuated both CXCR-associated chemotaxis genes, including *Cxcl1*, *Cxcl2*, *Cxcl6*, and *Cxcl9* and genes harboring interferon-stimulated responsive element (ISRE), such as *Mx1*, *Mx2*, *Oas1a*, *Oas2*, and *Cgas* (Figs. S4D and S4E). Additionally, we verified that upregulation of *Mx2*, an IFN-stimulated gene that mediates innate immunity, was inhibited by CQ treatment in aged kidney through RT-qPCR (Fig. S4F). We also performed an integrative comparison analysis of our transcription profiles with genes associated with kidney diseases and found that CQ-associated rescue DEGs correlated with the onset of a series of kidney diseases, such as renal fibrosis, nephritis, and chronic kidney failure (Fig. 2G). Thus, our data suggest that low-dose CQ treatment exhibits a beneficial effect in aged kidneys, potentially reducing innate immune response and improving ion transport function. Consistent with the beneficial effect of CQ on kidney, we found that CQ treatment downregulated genes associated with neutrophil chemotaxis, leukocyte migration and macrophage activation, while upregulated genes associated with fatty acid metabolic process, small molecule catabolic process and cellular catabolic process in aged liver (Fig. S4G), further indicating the potential hepato-protective effects of CQ.

Furthermore, we investigated the biological implications of pro-aging genes that were altered by CQ treatment. More than half of the pro-aging DEGs (1,678/2,934) were detected in the heart, most of which (1,594/1,678) were upregulated (Fig. S3A). By integrative analysis of pro-aging DEGs with genes associated with heart diseases, we found that CQ treatment-induced gene networks were associated with hypertrophic cardiomyopathy, cardiac arrhythmia, and heart failure (Fig. S4H). For instance, *Caps2*, which is also increased in patients with refractory ischemic end-stage heart failure, was further augmented by CQ treatment in aged heart (Fig. S4I). In addition, *Myh7*, a gene associated with cardiomyopathy, was upregulated in aged heart and further augmented by CQ (Fig. S4H). These data imply that despite seemingly normal heart function (Fig. 2C), low-dose CQ treatment may be associated with pro-aging side effects at the transcriptional level.

In this study, we found that low concentrations of CQ alleviated stem cell senescence, repressed tissue fibrosis, and extended lifespan (Fig. 2H). Multi-tissue transcriptomic inspection demonstrated that CQ may have both beneficial and detrimental effects on aged animals in a tissue-specific manner. By surveying the transcriptomic landscape of CQ-treated tissues, we found that low-dose CQ treatment attenuated age-associated gene expression across tissues. The strongest effect was observed in the kidney where we found decreased levels of ISRE-containing genes and increased expression of transporter encoding genes. However, CQ also augmented pro-aging transcriptional signatures, which may elicit potential cardiac toxicity without detectable functional impairment during the duration of the experiment. Our work systemically evaluates the phenotypic and transcriptional effects of CQ across mammalian tissues, thus providing a valuable resource to interrogate the potential effects of low-dose CQ usage.

Our transcriptomic profiling demonstrated the impact of CQ on 6 tissues in aged rats, and revealed tissue-specific effects of CQ. We identified aged kidneys as the most efficiently rescued tissue in terms of rescue DEGs. CQ treatment attenuated chronic inflammation and ISRE-containing genes, such as OAS/RNaseL, IFN, and cGAS. In addition, low-dose CQ treatment upregulated the genes involved in systemic ion homeostasis in the kidney and could at least partly rescue the transcriptomic signatures of multiple kidney diseases. Consistent with our results, CQ decreases renal fibrosis in chronic kidney diseases by inhibiting the lysosomal protease cathepsin D (Fox et al., 2016). However, CQ also had adverse effects on aged hearts at the transcriptional level. Most pro-aging DEGs were enriched in the heart, with an overrepresentation of cardiac disease-related gene sets. Consistently, the clinical cardiovascular toxicity of CQ has been reported (Wozniacka et al., 2006). For example, the treatment with chloroquine or hydroxychloroquine decreases myocardial cell discharge, leading to aberrant cardiac electrical conduction and a prolonged corrected QT interval based on electrocardiography (Wozniacka et al., 2006; Solomon and Lee, 2009). By contrast, although CQ has noticeable effects in hearts at the transcriptional level, extremely low-dose CQ may not affect normal heart function as assessed by transthoracic echocardiography and overall physiological health.

Notably, we found that low-dose CQ treatment extended the lifespan of naturally aged rats. The dose of CQ used in this study was only 0.1 mg/kg twice a week, at least 100-fold lower than the dose that was previously used in rodents (Solomon and Lee, 2009). Our results support the potential efficacy of CQ in delaying aging. Furthermore, it has been reported that 3.5 mg/kg of CQ enhanced DNA damage clearance and rescued age-related metabolic shift, suggesting a potential geroprotective effect (Qian et al., 2018). This lifespan extension effect by CQ was also observed recently in nematodes and a progeria mouse model (Qian et al., 2018). Our study shows that low-dose CQ extended the lifespan of naturally aged rodents.

The role of CQ in counteracting aging may be linked to its ability to inhibit chronic inflammation systematically and alleviate fibrosis. Consistent with our observations, CQ reduces inflammation and effectively decreases the salivary and serum levels of IL-6, a key component of SASP (Sargiacomo et al., 2020). In addition, CQ treatment is beneficial in alleviating mouse liver fibrosis induced by carbon tetrachloride (CCl_4_) via the inhibition of autophagy pathways (He et al., 2014). In paraquat-induced lung injury, CQ regulates oxidative stress, attenuates lung inflammation, and reduces fibrosis (Shen et al., 2017). Collectively, our data, together with others, indicate that CQ has beneficial roles in reducing chronic inflammation and tissue fibrosis, which could be harnessed to treat different age-related diseases.

In summary, our results demonstrate a geroprotective role of low-dose CQ on prematurely senescent human stem cells and physiologically aged rats. In general, these findings enable us to gain an in-depth understanding of the potential impact of CQ on organ aging and further emphasize the necessity of monitoring cardiac function when using CQ in clinical trials.

## Footnotes

The authors are grateful to Jingyi Jia (Xuanwu Hospital Capital Medical University), Xiang Shi, Xinyi Wu, Sai Yang, Jiajia Hou, Zhuanzhuan Xing, and Zheng Liu (Laboratory Animal Center, Institute of Biophysics, CAS) for the management of laboratory animals, Lei Bai, Qun Chu, Xiao Zhuo, Jing Lu, Ying Yang, Ruijun Bai, Luyang Tian and Shikun Ma for administrative assistance. This work was supported by the National Key Research and Development Program of China (2018YFC2000100), the Strategic Priority Research Program of the Chinese Academy of Sciences (XDA16000000), the Program of Beijing Municipal Science and Technology Commission (Z191100001519005), the National Natural Science Foundation of China (Grant Nos. 81921006, 81625009, 91749202, 81861168034, 91949209, 92049304, 81822018, 81870228, 81922027, 82071588, 92049116, 31801010, 81901433, 82125011, 82122024, 92149301, 92168201), the National Key Research and Development Program of China (2020YFA0804000, 2020YFA0112200, 2017YFA0103304, 2017YFA0102802, 2018YFA0107203, 2020YFA0113400), the Program of the Beijing Natural Science Foundation (Z190019, JQ20031), the Key Research Program of the Chinese Academy of Sciences (KFZD-SW-221), K. C. Wong Education Foundation (GJTD-2019-06, GJTD-2019-08), Beijing Hospitals Authority Youth Programme (QML20200802), Youth Innovation Promotion Association of CAS (E1CAZW0401, 2021078), the Non-profit Central Research Institute Fund of Chinese Academy of Medical Sciences (2020-JKCS-011), the State Key Laboratory of Stem Cell and Reproductive Biology, the State Key Laboratory of Membrane Biology, the 14th Five-year Network Security and Informatization Plan of Chinese Academy of Sciences (WX145XQ07-18), the Informatization Plan of Chinese Academy of Sciences (CAS-WX2021SF-0301), the Milky Way Research Foundation (MWRF), Young Elite Scientists Sponsorship Program by CAST (NO.YESS20200012), CAS Project for Young Scientists in Basic Research (YSBR-012) and the Tencent Foundation.

The authors declare no conflict of interest.

## Supplementary Information

Below is the link to the electronic supplementary material.

Supplementary file1 (PDF 9368 kb)

Supplementary file2 (XLSX 13 kb)
