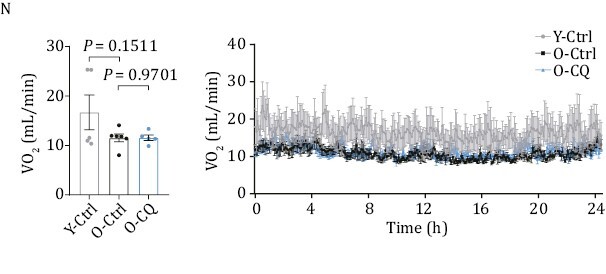# Correction to: Low-dose chloroquine treatment extends the lifespan of aged rats

**DOI:** 10.1093/procel/pwad053

**Published:** 2023-12-04

This is a correction to: Wei Li, Zhiran Zou, Yusheng Cai, Kuan Yang, Si Wang, Zunpeng Liu, Lingling Geng, Qun Chu, Zhejun Ji, Piu Chan, Guang-Hui Liu, Moshi Song, Jing Qu, Weiqi Zhang, Low-dose chloroquine treatment extends the lifespan of aged rats, Protein & Cell, Volume 13, Issue 6, June 2022, Pages 454–461, https://doi.org/10.1007/s13238-021-00903-1

The authors wish to introduce the following corrections to their article.

Figure 1N showed real-time measurement of oxygen consumption rate (VO_2_, mL/min) of Y-Ctrl, O-Ctrl and O-CQ rats by metabolic cage detection, where the Y-axis of the plots was mistakenly labelled as “VCO_2_” instead of the intended “VO_2_”. A new Figure 1N is provided below. The correction does not affect any result, conclusion, or discussion of this study. These details have been corrected only in this correction notice to preserve the published version of record.

Figure 1N