# Supplementary material for: Low-dose chloroquine treatment extends the lifespan of aged rats
Source: Protein Cell. 2022 Jan 12;13(6):454–61. doi: 10.1007/s13238-021-00903-1 (PMC9095792; doi:10.1007/s13238-021-00903-1)
Supplement: Supplementary file 1 — Supplementary file1 (PDF 9368 kb) [file 13238_2021_903_MOESM1_ESM.pdf]

Supplemental Materials

Materials and Methods

Cell culture

WS hMSCs (Werner syndrome hMSCs) were cultured on a gelatin-coated plate (Zhang et al., 2015; Wu et al., 2018). The hMSC medium contains α-MEM medium, supplemented with 10% fetal bovine serum (FBS, Gibco), 0.1 mM non-essential amino acids (NEAA, Gibco), 1% penicillin/streptomycin (Gibco), and 1 ng/mL bFGF (Joint Protein Central). WS hMSCs were seeded in 6-well plates at a density of 8×10^4 cells and in 96-well plates at a density of 2500 cells per well. After a 24 h culturing period, CQ (Sigma, C6628) (0.2 μmol/L, 1 μmol/L, 5 μmol/L, 20 μmol/L, 100 μmol/L) was added to the medium. No mycoplasma contamination was detected in the cell culture.

Drug treatment

All animal experiments were performed in accordance with the protocol approved by the Animal Care and Use Committee of the Chinese Academy of Sciences. Wild-type SD rats were individually reared in a pathogen-free facility at a controlled temperature (23-24°C), under a 12 h light and dark cycle with food and water provided *ad libitum*. The cage and bedding were changed once a week. For CQ treatment, rats were treated at 0.1 mg of CQ per kilogram of body weight. Before the experiment, the lowest water intake of the rats was measured to ensure that the given drug was consumed by the rat. Accordingly, CQ (0.1 mg/kg) was dissolved in 3 mL of water and given to each rat at 8:00 am on the day of administration. Only after having finished the 3 mL water with CQ in the bottle, the rats were then provided drinking water *ad libitum*. Male SD rats (24-month-old) were treated with CQ twice per week for five months. Water was provided to the old control group starting from 24-month-old in the same way without CQ. In addition, 2-month-old young rats were taken as young controls. Body weight and blood glucose of the rats were measured monthly. After 5 months of CQ treatment, rats were tested in a metabolic cage and by cardiac echocardiography and samples were collected for further analyses. Male SD rats were treated with CQ twice per week until the death in the lifespan experiment.

Metabolic cage detection

Metabolism cage detection was performed with rats treated with CQ or water (control) as

previously described (Campos et al., 2017; Dodd et al., 2017) by Promethion Small Animal Metabolism Measurement and Analysis System (Sable Systems). The macro interpreter program was used to convert the recorded data file format into excel files after the experiment for further statistical analyses.

Routine blood analysis

Blood biochemistry in freshly collected whole blood of rats was tested using the hematology analyzer (Shenzhen Mindray BC-5180CRP). The experiment was carried out in accordance with the manufacturer's instructions.

Liver function test

Blood biochemistry in the plasma of rats was performed using the fully automatic bio analysis machine (Toshiba, TBA-120FR), and then subjected to several tests by using the kits listed below. ALT (Beijing XinChuangYuan Biotech, B2008) and TBiL (Beijing XinChuangYuan Biotech, B2081) were measured following the manufacturer’s instructions.

Assessment of cardiac function

The cardiac function was evaluated through transthoracic echocardiography by Vevo 2100 imaging system (Visual Sonics, Inc.) with a 30-MHz transducer. The mice were anesthetized via the inhalation of 2% isoflurane and then tested. The two-dimensional M-mode trace of each rat was obtained at the level of the papillary muscle. Left ventricular ejection fraction (EF), fractional shortening (FS) and E/A ratios were measured and calculated on three consecutive cardiac cycles.

Enzyme-linked immunosorbent assay (ELISA)

Pro-inflammatory cytokine levels in the plasma of rats were tested by enzyme-linked immunosorbent assay (ELISA, bioscience). IL-6 (Thermo, 88-50625), TNF-α (Thermo, 88-7340), MCP-1 (Thermo, BMS631), IL-1β (Thermo, 88-6010) and IL-7 (Thermo, ERA33RB) were tested in accordance with the manufacturer's instructions. The results were quantified using a microplate reader (Thermo, MK3). IL-6 levels in the cell supernatant of WS hMSCs were measured by a commercially available ELISA kit (Biolegend, 430504) according to the manufacturer’s instructions.

Preparation of paraffin sections and frozen sections

Paraffin embedding and frozen sectioning were conducted as previously described (Ma et al., 2020). For paraffin-embedded sections, fresh rat kidney, lung, liver, heart, aorta, and small intestine samples were collected and immersed in 4% paraformaldehyde (PFA) at 4°C overnight. The samples were then dehydrated with gradient alcohol, embedded in paraffin, and cut into 5-μm sections using the rotary microtome HM325 (MICROM International). For frozen sections, rat samples were embedded in an optimal cutting temperature (OCT) compound (Sakura Finetek, 4583), embedded at room temperature for 15 min, quickly frozen in liquid nitrogen, and then stored at -80°C. ACM 1850 cryostat (Leica Microsystems Nussloch) was used to cut the frozen samples into 10-μm sections and stored at -80°C.

Immunofluorescence staining

Immunofluorescence microscopy was performed as previously described (Ma et al., 2020). For tissues, paraffin-embedded sections of rat tissue samples were routinely deparaffinized to water, and antigen retrieval was performed by boiling in sodium citrate buffer (pH = 6.0) for 15 min. After cooling down to room temperature, the sections were blocked with 5% donkey serum in PBS for 1 h at room temperature. After washing, samples were incubated with the primary antibody Anti-CD68 (Abcam, ab213363) in blocking buffer at 4°C overnight. The next day, the appropriate fluorescently labeled secondary antibody was added. Sections were subsequently counterstained with Hoechst 33342 (Thermo Fisher Scientific). The image was taken using a Zeiss LSM900 laser scanning confocal microscope, and statistical data were calculated and analyzed using ImageJ.

For cells, samples were seeded on microscope coverslips for 24 h before the staining. After being washed with PBS twice, cell slides were fixed with 4% paraformaldehyde in PBS for 15 min. Later, they were washed with PBS three times, followed by cell permeabilization with 0.3% Triton X-100 in PBS for 20 min. Blocking buffer (5% donkey serum in PBS) was then applied before the incubation with appropriate primary antibody (anti-Ki67 (Abcam, ab16667), anti-H3K9me3 (Abcam, ab8898), anti-HP1γ (CST, 2619S) or anti-LAP2 (Abcam, ab5162)) in blocking buffer at 4°C overnight followed by the incubation with appropriate fluorescently labeled secondary antibody and Hoechst 33342. All the images were obtained using a Zeiss LSM900 laser scanning confocal microscope.

Aggresome staining

For analysis of aggresome intensity, frozen sections were stained with PROTEOSTAT®

aggresome detection kit (ENZO, ENZ-51035-K100). Briefly, sections were fixed using 4% PFA in PBS for 15 min, permeabilized with 0.3% Triton X-100 (Sigma, T9284) in PBS for 7 min, incubated with the aggresome dye (1:2000 dilution in PBS) for 3 min, and destained in 1% acetic acid for 20 min. After washing thoroughly with PBS, sections were subsequently counterstained with Hoechst 33342 (Thermo Fisher Scientific). The image was taken using a Zeiss LSM900 laser scanning confocal microscope and statistical data were calculated and analyzed using ImageJ.

Hematoxylin and eosin (H&E) staining

Paraffin-embedded sections were routinely deparaffinized in water and incubated with hematoxylin (Servicebio, China) at room temperature for 5 min, followed by treatment with 1% acidic alcohol for less than 10 sec as appropriate. Next, slides were washed in running tap water and then stained with eosin. The sections were dehydrated through 80%, 90% and 100% alcohol, and cleared by xylene. Slides were covered with coverslips and images were taken for quantitative analysis.

Masson's trichrome staining

Masson's trichrome staining (Solarbio, G1346) was performed according to the protocol. The paraffin-embedded sections of the tested tissues were deparaffinized using xylene and rehydrated with 100% alcohol, 95% alcohol, 70% alcohol, and running tap water. Sections were then stained according to the manufacturer's protocol. The image was taken using the slice scanner (Leica, CS2) for quantitative analysis.

SA-β-gal staining

SA-β-gal staining was conducted as previously described (Cai et al., 2020). For tissues, frozen sections were warmed up to room temperature, washed with PBS, and fixed with 2% formaldehyde and 0.2% glutaraldehyde at room temperature for 15 min. Tissue sections were then stained with freshly prepared 1 mg/mL of X-gal solution (Ameresco) at 37°C for 4-12 h, depending on the tissue type. For cells, cells were seeded on 6-well plates for 24 h before staining. After being washed by PBS twice, cell slides were fixed with 2% formaldehyde and 0.2% glutaraldehyde at room temperature for 4 min. After washing with PBS for three times, the samples were stained in freshly prepared 1 mg/mL of X-gal solution at 37°C overnight. Images were collected under a microscope and the percentages of SA-β-gal-positive areas and cells were quantified using ImageJ.

RT-qPCR

Total RNAs from different tissues were extracted by TRIzol (Gibco, 15596018,) and converted to cDNA using GoScript Reverse Transcription System (Promega, A5001). RT-qPCR was performed using SYBR qPCR mix (TOYOBO, QPS-201) on a CFX384-Real-time system (Bio-Rad). *Actin* was used as an internal control to normalize the expression of target genes. Primers used for RT-qPCR are listed in the Table S1.

RNA sequencing

An equal amount of extracted RNAs from each rat of the same group was mixed and then was applied for RNA sequencing with three technical replicates. Although this mixed procedure may cover the heterogeneity between rats, this method helps to identify key transcriptome changes between groups. Transcriptome library construction was performed with NEBNext Ultra™ Directional RNA Library Prep Kit (NEB, USA). The generated library was sequenced on an Illumina platform by the Novogene Bioinformatics Technology.

RNA-seq data processing

Raw data were trimmed with Trim Galore software (version 0.4.5) (https://github.com /FelixKrueger/TrimGalore) to remove low-quality reads and reads with adaptors. The cleaned reads were then mapped to the UCSC rat reference genome (rn6) using HISAT2 (version 2.2.1) (Kim et al., 2015). Expression levels for each annotated gene were calculated by HTSeq (version 0.12.4) (Anders et al., 2015). Differentially expressed genes (DEGs) were calculated using DESeq2 package (version 1.30.0) (Love et al., 2014) with the cutoff of Benjamin-Hochberg adjusted *P*-value < 0.05 and $|\log_2(\text{fold change})| > 0.5$. GO term and pathway enrichment analysis was performed using Metascape (http://metascape.org) (Zhou et al., 2019). Gene set enrichment analysis was conducted using GSEA (version 4.1.0) with default parameters (Subramanian et al., 2007). The aging-related genes were downloaded from the Aging Atlas database (https://bigd.big.ac.cn/aging/index) and the specific disease-associated genes were obtained from DisGeNET (https://www.disgenet.org) and MalaCards (https://www.malacards.org). All the sequencing data are deposited in Genome Sequence Archive (GSA) (https://bigd.big.ac.cn/gsa/) with the accession number of CRA004738.

Statistical analysis

Data are presented as means ± SEM. Statistical analyses were performed using GraphPad Prism

(v8) with the unpaired Student's *t*-test with post-hoc test or Log-rank test as appropriate. *P*-values less than 0.05 were considered statistically significant.

Supplemental Figure Legends

Figure S1. Functional analyses of aging and CQ-treated rats.

(A) Immunostaining of H3K9me3, HP1γ and LAP2 in CQ-treated and control WS hMSCs. Scale bar, 25 μm. White arrows indicate the cells with decreased H3K9me3, HP1γ and LAP2 expression. Data are shown as means ± SEM. $n > 300$ cells from three biological replicates (unpaired Student's *t*-test). (B) ELISA of IL-6, MCP-1, IL-7 and IL-1β in the plasma of Y-Ctrl, O-Ctrl and O-CQ rats. The data are shown as means ± SEM. $n = 5$ rats per group (unpaired Student's *t*-test). IL, interleukin; MCP-1, monocyte chemoattractant protein-1. (C) Monthly monitoring of body weight (g) and blood glucose concentration (mmol/L) of O-Ctrl and O-CQ rats during CQ treatment. The data are shown as means ± SEM. $n = 5$ rats per group (unpaired Student's *t*-test). (D) Organ weights of brain, heart, liver, lung and kidney in Y-Ctrl, O-Ctrl and O-CQ rats. The data are shown as means ± SEM. $n = 5$ rats per group (unpaired Student's *t*-test). (E) The tissue index (tissue weight divided by body weight) of heart, liver, lung and kidney of Y-Ctrl, O-Ctrl and O-CQ rats. The data are shown as means ± SEM. $n = 5$ rats per group (unpaired Student's *t*-test).

Figure S2. Morphological analyses of aging and CQ-treated rats.

(A) H&E staining of kidney, small intestine, heart and aorta tissues. Scale bars, 50 μm for kidney and heart, 200 μm for small intestine and 200 μm for aorta. The yellow line indicates the edge of the glomerulus in the kidney tissue, the length of the intestinal villi in the intestinal tissue, the edge of the myocardial fiber area in the heart tissue, and the thickness of the aortic wall in the aortic tissue. Quantitative data are shown as means ± SEM. $n = 5$ rats per group (unpaired Student's *t*-test). (B) Immunostaining for a macrophage marker CD68 in the liver. Scale bar, 50 μm. White arrows indicate CD68-positive cells. The data are shown as means ± SEM. $n = 5$ rats per group. Over 300 cells were quantified per individual (unpaired Student's *t*-test). (C) Aggresome staining in kidney and liver tissues of Y-Ctrl, O-Ctrl, O-CQ-treated rats. Scale bars, 30 μm for kidney and liver. White arrows indicate aggresome-positive cells. Quantitative data are shown as means ± SEM. $n = 5$ rats per group (unpaired Student's *t*-test). (D) SA-β-gal staining of kidney, liver, heart and lung tissues. Scale bars, 100 μm for kidney, liver and lung. Quantitative data are shown as means ± SEM. $n = 5$ rats per group (unpaired Student's *t*-test).

Figure S3. Transcriptional characterization of aging and CQ-treated rats.

(A) Heatmaps showing the distribution of rescue DEGs (green), failure to rescue DEGs (dark gray), CQ-specific DEGs (orange) and pro-aging DEGs (brown) in the six tissues including kidney, small intestine, liver, heart, lung and aorta. Each column represents one gene. Red, upregulated gene (Log_2FC [log_2(fold change)] > 0.5, adjusted *P*-value < 0.05); blue, downregulated gene (Log_2FC < -0.5, adjusted *P*-value < 0.05); gray, unchanged gene ($|Log_2FC|$ < 0.5 or adjusted *P*-value > 0.05). (B) Rose charts showing the numbers of aging DEGs, CQ DEGs, rescue DEGs, failure to rescue DEGs and pro-aging DEGs in the six tissues. Red indicates upregulated genes and blue indicates downregulated genes. (C) Gene set enrichment analysis (GSEA) plots showing decreased IFN response in kidney, liver, heart and aorta upon CQ treatment. (D) GSEA plots showing decreased SASP in kidney, liver and aorta upon CQ treatment. (E) GSEA plot showing decreased IL-6-JAK-STAT3 signaling in liver upon CQ treatment. (F) GSEA plot showing decreased TGF-β signaling in liver upon CQ treatment. (G) Heatmap showing the shared genes between downregulated rescue DEGs and aging-associated genes in Aging Atlas database among the six tissues.

Figure S4. Rescue effects and pro-aging changes at the transcriptional level upon CQ treatment.

(A) Heatmap showing the upregulated and downregulated rescue DEGs shared by at least three tissues upon CQ treatment. (B) Representative GO terms and pathways enriched in rescue DEGs in kidney. (C) Heatmap showing changes in *Slc24a3*, *Slc17a2*, *Slc31a1* and *Slc36a4* upon CQ treatment in kidney. Red indicates upregulated, and blue indicates downregulated. (D) Heatmaps showing rescue DEGs associated with an interferon-stimulated responsive element (ISRE) and CXCR chemokine upon CQ treatment in kidney. Red indicates upregulated genes and blue indicates downregulated genes. (E) GSEA plot showing decreased chemokine receptors in kidney upon CQ treatment. (F) RT-qPCR analysis verifying the changes of top rescued DEGs in kidney. Data are shown as the means ± SEM. n = 5 rats per group (unpaired Student's *t*-test). (G) Representative GO terms and pathways enriched in rescue DEGs in liver. (H) Network plot showing pro-aging DEGs associated with cardiac diseases in heart. (I) RT-qPCR analysis verifying the changes of top pro-aging DEGs in heart. Data are shown as the means ± SEM. n = 4-5 rats per group (unpaired Student's *t*-test).

Supplemental Table Legends

Table S1. RT-qPCR primer lists.

References

Anders, S., Pyl, P.T., and Huber, W. (2015). HTSeq-a Python framework to work with high-throughput sequencing data. Bioinformatics 31, 166-169.

Cai, Y., Zhou, H., Zhu, Y., Sun, Q., Ji, Y., Xue, A., Wang, Y., Chen, W., Yu, X., Wang, L., et al. (2020). Elimination of senescent cells by beta-galactosidase-targeted prodrug attenuates inflammation and restores physical function in aged mice. Cell Res 30, 574-589.

Campos, C.A., Bowen, A.J., Han, S., Wisse, B.E., Palmiter, R.D., and Schwartz, M.W. (2017). Cancer-induced anorexia and malaise are mediated by CGRP neurons in the parabrachial nucleus. Nature neuroscience 20, 934-942.

Dodd, G.T., Andrews, Z.B., Simonds, S.E., Michael, N.J., DeVeer, M., Brüning, J.C., Spanswick, D., Cowley, M.A., and Tiganis, T. (2017). A Hypothalamic Phosphatase Switch Coordinates Energy Expenditure with Feeding. Cell metabolism 26, 577.

Ma, S., Sun, S., Geng, L., Song, M., Wang, W., Ye, Y., Ji, Q., Zou, Z., Wang, S., He, X., et al. (2020). Caloric Restriction Reprograms the Single-Cell Transcriptional Landscape of Rattus Norvegicus Aging. Cell 180, 984-1001 e1022.

Subramanian, A., Kuehn, H., Gould, J., Tamayo, P., and Mesirov, J.P. (2007). GSEA-P: a desktop application for Gene Set Enrichment Analysis. Bioinformatics (Oxford, England) 23, 3251-3253.

Wu, Z., Zhang, W., Song, M., Wang, W., Wei, G., Li, W., Lei, J., Huang, Y., Sang, Y., Chan, P., et al. (2018). Differential stem cell aging kinetics in Hutchinson-Gilford progeria syndrome and Werner syndrome. Protein & cell 9, 333-350.

Zhang, W., Li, J., Suzuki, K., Qu, J., Wang, P., Zhou, J., Liu, X., Ren, R., Xu, X., Ocampo, A., et al. (2015). A Werner syndrome stem cell model unveils heterochromatin alterations as a driver of human aging. Science (New York, NY) 348, 1160-1163.

Zhou, Y., Zhou, B., Pache, L., Chang, M., Khodabakhshi, A.H., Tanaseichuk, O., Benner, C., and Chanda, S.K. (2019). Metascape provides a biologist-oriented resource for the analysis of systems-level datasets. Nat Commun 10, 1523.

Figure S1

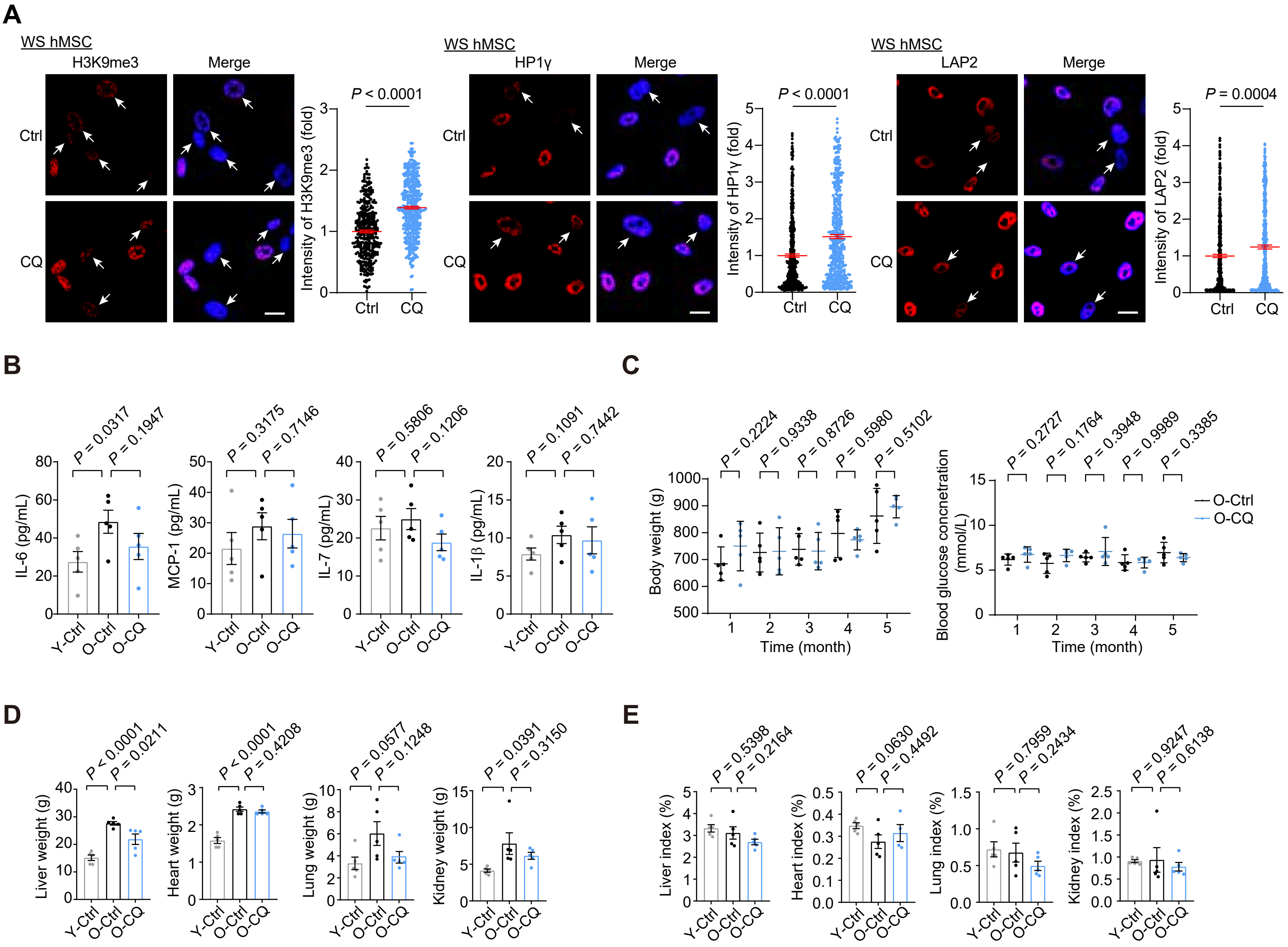
A
WS hMSC
H3K9me3
Merge
Ctrl
CQ
P < 0.0001
Intensity of H3K9me3 (fold)
HP1γ
Intensity of HP1γ (fold)
LAP2
P = 0.0004
Intensity of LAP2 (fold)
B
P = 0.0317
P = 0.1947
IL-6 (pg/mL)
P = 0.3175
P = 0.7146
MCP-1 (pg/mL)
P = 0.5806
P = 0.1206
IL-7 (pg/mL)
P = 0.1091
P = 0.7442
IL-1β (pg/mL)
Y-Ctrl
O-Ctrl
O-CQ
C
P = 0.2224
P = 0.9338
P = 0.8726
P = 0.5980
P = 0.5102
Body weight (g)
Time (month)
P = 0.2727
P = 0.1764
P = 0.3948
P = 0.9989
P = 0.3385
Blood glucose concnetration (mmol/L)
O-Ctrl
O-CQ
D
P < 0.0001
P = 0.0211
Liver weight (g)
P < 0.0001
P = 0.4208
Heart weight (g)
P = 0.0577
P = 0.1248
Lung weight (g)
P = 0.0391
P = 0.3150
Kidney weight (g)
E
P = 0.5398
P = 0.2164
Liver index (%)
P = 0.0630
P = 0.4492
Heart index (%)
P = 0.7959
P = 0.2434
Lung index (%)
P = 0.9247
P = 0.6138
Kidney index (%)

Figure S2

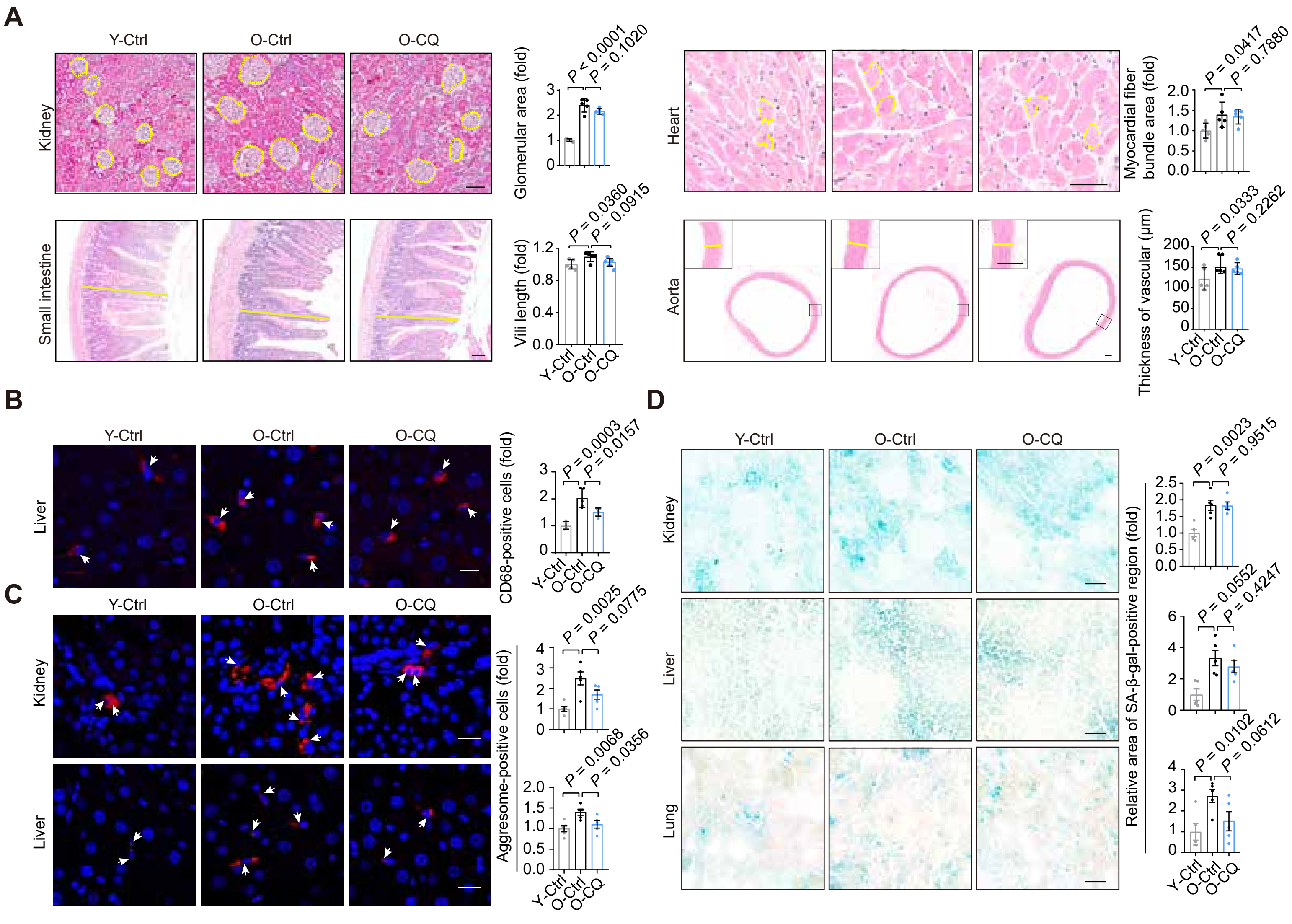
A
Y-Ctrl
O-Ctrl
O-CQ
Kidney
Small intestine
Glomerular area (fold)
P < 0.0001
P = 0.1020
Vili length (fold)
P = 0.0360
P = 0.0915
Heart
Aorta
Myocardial fiber bundle area (fold)
P = 0.0417
P = 0.7880
Thickness of vascular (μm)
P = 0.0333
P = 0.2262
B
Liver
CD68-positive cells (fold)
P = 0.0003
P = 0.0157
C
Aggresome-positive cells (fold)
P = 0.0025
P = 0.0775
P = 0.0068
P = 0.0356
D
Lung
Relative area of SA-β-gal-positive region (fold)
P = 0.0023
P = 0.9515
P = 0.0552
P = 0.4247
P = 0.0102
P = 0.0612

Figure S3

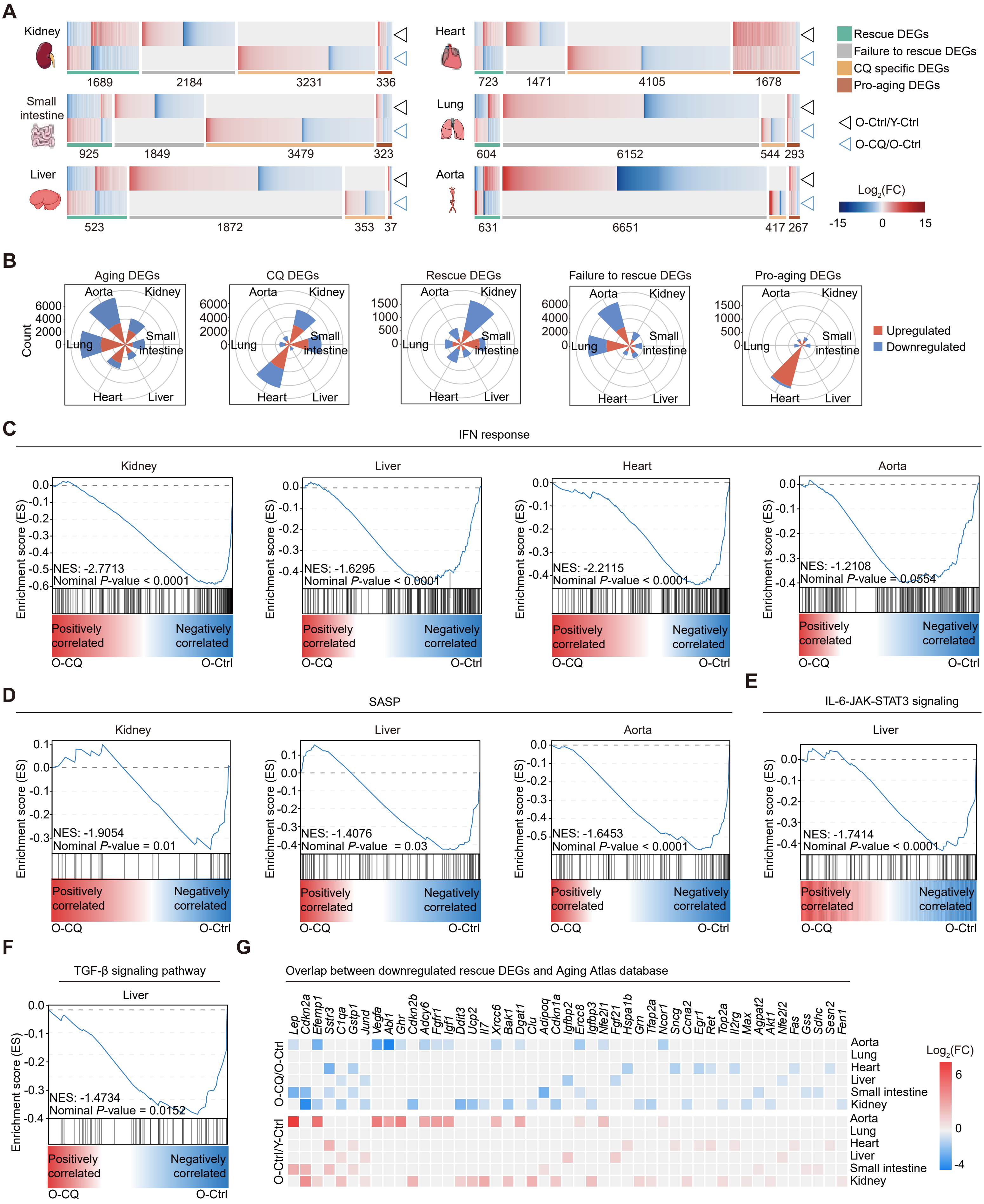
A
Kidney
1689
2184
3231
336
Heart
723
1471
4105
1678
Small intestine
925
1849
3479
323
Lung
604
6152
544
293
Liver
523
1872
353
37
Aorta
631
6651
417
267
Rescue DEGs
Failure to rescue DEGs
CQ specific DEGs
Pro-aging DEGs
O-Ctrl/Y-Ctrl
O-CQ/O-Ctrl
Log2(FC)
-15
0
15
B
Aging DEGs
CQ DEGs
Rescue DEGs
Failure to rescue DEGs
Pro-aging DEGs
Count
Aorta
Kidney
Lung
Small intestine
Heart
Liver
Upregulated
Downregulated
C
IFN response
Kidney
NES: -2.7713
Nominal P-value < 0.0001
Liver
NES: -1.6295
Nominal P-value < 0.0001
Heart
NES: -2.2115
Nominal P-value < 0.0001
Aorta
NES: -1.2108
Nominal P-value = 0.0554
Enrichment score (ES)
Positively correlated
Negatively correlated
O-CQ
O-Ctrl
D
SASP
Kidney
NES: -1.9054
Nominal P-value = 0.01
Liver
NES: -1.4076
Nominal P-value = 0.03
Aorta
NES: -1.6453
Nominal P-value < 0.0001
E
IL-6-JAK-STAT3 signaling
Liver
NES: -1.7414
Nominal P-value < 0.0001
F
TGF-β signaling pathway
Liver
NES: -1.4734
Nominal P-value = 0.0152
G
Overlap between downregulated rescue DEGs and Aging Atlas database
Lep Cdkn2a Efemp1 Sstr3 C1qa Gstp1 Jund Vegfa Abl1 Ghr Cdkn2b Adcy6 Fgfr1 Igf1 Ddit3 Ucp2 Il7 Xrcc6 Bak1 Dgat1 Clu Adipoq Cdkn1a Igfbp2 Ercc8 Igfbp3 Nfe2l1 Fgf21 Hspa1b Grn Tfap2a Ncor1 Sncg Ccna2 Egr1 Ret Top2a Il2rg Max Agpat2 Akt1 Nfe2l2 Fas Gss Sdhc Sesn2 Fen1
O-CQ/O-Ctrl
O-Ctrl/Y-Ctrl
Aorta
Lung
Heart
Liver
Small intestine
Kidney
Log2(FC)
6
0
-4

Figure S4

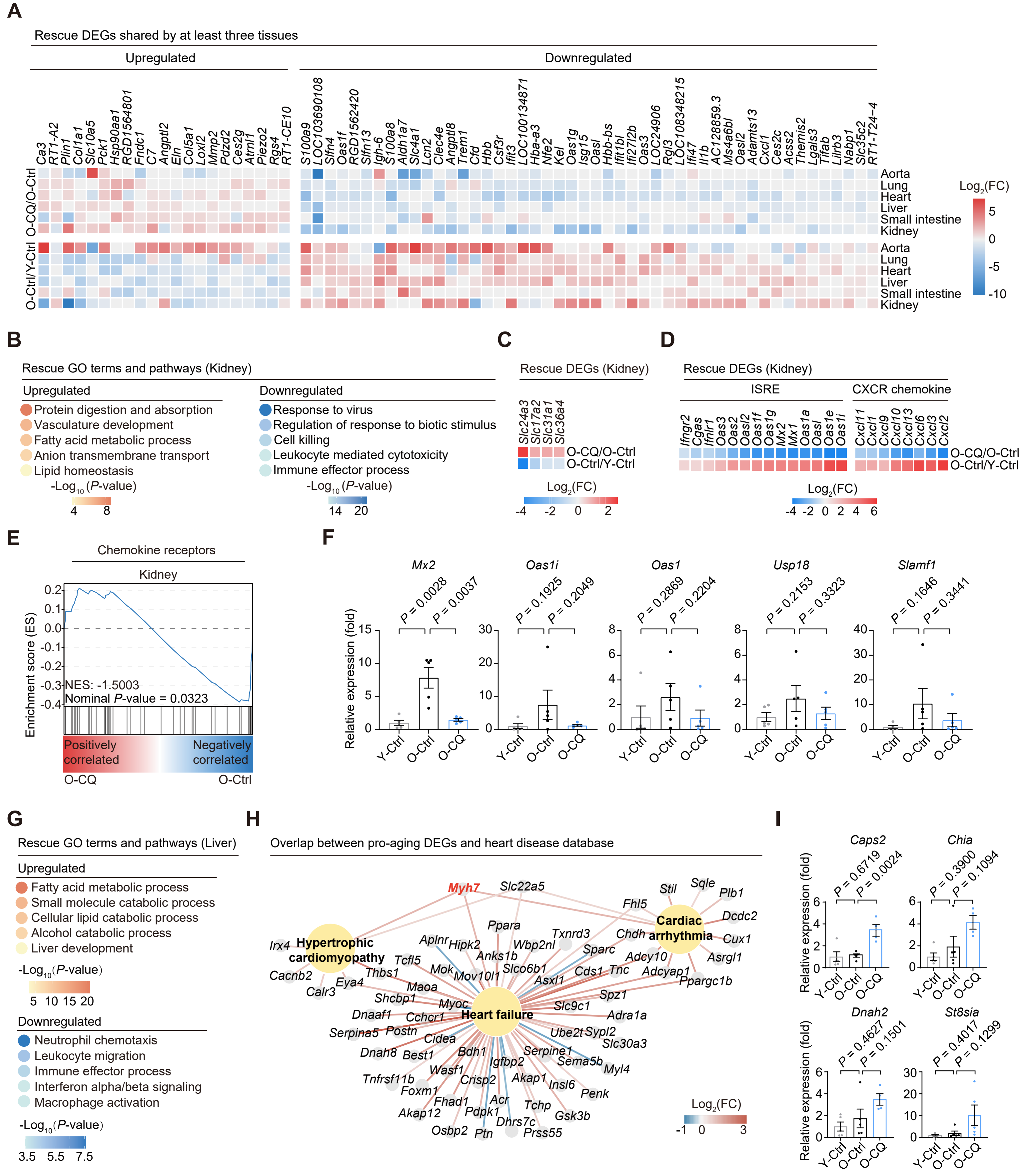
A
Rescue DEGs shared by at least three tissues
Upregulated
Downregulated
Ca3 RT1-A2 Plin1 Col1a1 Slc10a5 Pck1 Hsp90aa1 RGD1564801 Fndc1 C7 Angptl2 Eln Col5a1 Loxl2 Mmp2 Pdzd2 Ces2g Atrnl1 Piezo2 Rgs4 RT1-CE10
S100a9 LOC103690108 Slfn4 Oas1f RGD1562420 Slfn13 Igh-6 S100a8 Aldh1a7 Slc4a1 Lcn2 Clec4e Angptl8 Trem1 Cfd Hbb Csf3r Ifit3 LOC100134871 Hba-a3 Nfe2 Kel Oas1g Isg15 Oasl Hbb-bs Ifit1bl Ifi27l2b Oas3 LOC24906 Rgl3 LOC108348215 Ifi47 Il1b AC128859.3 Ms4a6bl Oasl2 Adamts13 Cxcl1 Ces2c Acss2 Themis2 Lgals3 Tfab Lilrb3 Nabp1 Slc35c2 RT1-T24-4
O-CQ/O-Ctrl
O-Ctrl/Y-Ctrl
Aorta
Lung
Heart
Liver
Small intestine
Kidney
Log2(FC)
5
0
-5
-10
B
Rescue GO terms and pathways (Kidney)
Upregulated
Protein digestion and absorption
Vasculature development
Fatty acid metabolic process
Anion transmembrane transport
Lipid homeostasis
-Log10(P-value)
4 8
Downregulated
Response to virus
Regulation of response to biotic stimulus
Cell killing
Leukocyte mediated cytotoxicity
Immune effector process
-Log10(P-value)
14 20
C
Rescue DEGs (Kidney)
Slc24a3 Slc17a2 Slc31a1 Slc36a4
O-CQ/O-Ctrl
O-Ctrl/Y-Ctrl
Log2(FC)
-4 -2 0 2
D
Rescue DEGs (Kidney)
ISRE
CXCR chemokine
Ifngr2 Cgas Ifnlr1 Oas3 Oas2 Oasl2 Oas1f Oas1g Mx2 Mx1 Oas1a Oasl Oas1e Oas1i
Cxcl11 Cxcl1 Cxcl9 Cxcl10 Cxcl13 Cxcl6 Cxcl3 Cxcl2
O-CQ/O-Ctrl
O-Ctrl/Y-Ctrl
Log2(FC)
-4 -2 0 2 4 6
E
Chemokine receptors
Kidney
Enrichment score (ES)
NES: -1.5003
Nominal P-value = 0.0323
Positively correlated
Negatively correlated
O-CQ
O-Ctrl
F
Mx2
P = 0.0028
P = 0.0037
Oas1i
P = 0.1925
P = 0.2049
Oas1
P = 0.2869
P = 0.2204
Usp18
P = 0.2153
P = 0.3323
Slamf1
P = 0.1646
P = 0.3441
Relative expression (fold)
Y-Ctrl
O-Ctrl
O-CQ
G
Rescue GO terms and pathways (Liver)
Upregulated
Fatty acid metabolic process
Small molecule catabolic process
Cellular lipid catabolic process
Alcohol catabolic process
Liver development
-Log10(P-value)
5 10 15 20
Downregulated
Neutrophil chemotaxis
Leukocyte migration
Immune effector process
Interferon alpha/beta signaling
Macrophage activation
-Log10(P-value)
3.5 5.5 7.5
H
Overlap between pro-aging DEGs and heart disease database
Hypertrophic cardiomyopathy
Cardiac arrhythmia
Heart failure
Myh7
Slc22a5
Stil
Sqle
Plb1
Fhl5
Dcdc2
Ppara
Txnrd3
Chdh
Cux1
Irx4
Aplnr
Hipk2
Wbp2nl
Sparc
Asrgl1
Tcfl5
Anks1b
Adcy10
Cacnb2
Thbs1
Mok
Slco6b1
Cds1
Tnc
Adcyap1
Eya4
Mov10l1
Asxl1
Ppargc1b
Calr3
Maoa
Spz1
Shcbp1
Myoc
Slc9c1
Dnaaf1
Cchcr1
Adra1a
Serpina5
Postn
Ube2t
Sypl2
Cidea
Slc30a3
Dnah8
Best1
Bdh1
Serpine1
Igfbp2
Sema5b
Myl4
Wasf1
Tnfrsf11b
Crisp2
Akap1
Insl6
Foxm1
Penk
Fhad1
Acr
Tchp
Akap12
Pdpk1
Gsk3b
Dhrs7c
Osbp2
Ptn
Prss55
Log2(FC)
-1 0 3
I
Caps2
P = 0.6719
P = 0.0024
Chia
P = 0.3900
P = 0.1094
Dnah2
P = 0.4627
P = 0.1501
St8sia
P = 0.4017
P = 0.1299
Relative expression (fold)
Y-Ctrl
O-Ctrl
O-CQ
